# Penetrating ocular trauma associated with blank cartridge

**DOI:** 10.1186/1471-2415-14-23

**Published:** 2014-03-03

**Authors:** Sunghyuk Moon, Su-Ho Lim

**Affiliations:** 1 Department of Ophthalmology, Inje University Busan Paik Hospital, Busan, Republic of Korea; 2 Department of Ophthalmology, Yeungnam University College of Medicine, Daegu, Republic of Korea; 3 Department of Ophthalmology, Daegu Veterans Health Service Medical Center, 60 Wolgok-ro, Dalseo-Gu, Daegu 704-802, Republic of Korea

**Keywords:** Blank ammunition, Blank cartridge, Ocular trauma, Ultrasonography

## Abstract

**Background:**

Blank cartridge guns are generally regarded as being harmless and relative safe. However recent published articles demonstrated that the gas pressure from the exploding propellant of blank cartridge is powerful enough to penetrate the thoracic wall, abdominal muscle, small intestine and the skull. And there has been a limited number of case reports of ocular trauma associated with blank cartridge injury. In addition, no report on case with split extraocular muscle injury with traumatic cataract and penetrating corneoscleral wound associated with blank cartridge has been previously documented. This report describes the case of patient who sustained penetrating ocular injury with extraocular muscle injury by a close-distance blank cartridge that required surgical intervention.

**Case presentation:**

A 20-year-old man sustained a penetrating globe injury in the right eye while cleaning a blank cartridge pistol. His uncorrected visual acuity at presentation was hand motion and he had a flame burn of his right upper and lower lid with multiple missile wounds. On slit-lamp examination, there was a 12-mm laceration of conjunctiva along the 9 o'clock position with two pinhole-like penetrating injuries of cornea and sclera. There was also a 3-mm corneal laceration between 9 o'clock and 12 o'clock and the exposed lateral rectus muscle was split. Severe Descemet's membrane folding with stromal edema was observed, and numerous yellow, powder-like foreign bodies were impacted in the cornea. Layered anterior chamber bleeding with traumatic cataract was also noted. Transverse view of ultrasonography showed hyperechoic foreign bodies with mild reduplication echoes and shadowing. However, a computed tomographic scan using thin section did not reveal a radiopaque foreign body within the right globe.

**Conclusion:**

To our best knowledge, this is the first case report of split extraocular muscle injury with traumatic cataract and penetrating ocular injury caused by blank cartridge injury. Intraocular foreign bodies undetectable by CT were identified by B-scan ultrasonography in our patient. This case highlights the importance of additional ultrasonography when evaluating severe ocular trauma. And ophthalmologists should consider the possibility of penetrating injury caused by blank ammunition.

## Background

Blank cartridge is special type of ammunition, and its purpose is mainly the sound imitation of shooting [1,2]. Blank cartridge is widely used for shooting practice, for start guns, or in theaters etc. [1,3]. Blank cartridge guns are generally regarded as being harmless and relative safe [3] and are not considered to be firearms in legal sense in most countries [4]. Thus, blank cartridge can be purchased by adults due to lack of legal regulation in some European countries [5].

Recent published articles demonstrated that the gas pressure from the exploding propellant of blank cartridge is powerful enough to penetrate the thoracic wall, abdominal muscle, small intestine and the skull [3,5]. In addition, Uner et al. [6] reported that a 9 mm blank cartridge is possible to penetrate a 0.5 cm thick, piece of poly-wood. In this context, the potential of blank cartridge to inflict serious and potentially lethal injuries is still grossly underestimated [5].

However, there has been a limited number of case reports of ocular trauma associated with blank cartridge injury. Previous articles reported that corneal foreign bodies were chemically inert and do not excite an inflammatory reaction [7,8]. Even though, Runyan and Ewald reported that blank cartridge did not penetrate the ocular surface and epithelial damage and endothelial edema resolved in 24–72 hours without apparent residual effects [7].

In addition, no report on case with split extraocular muscle injury with traumatic cataract and penetrating corneoscleral wound associated blank cartridge has been previously documented. We herein report the case of 20-year-old patient who sustained penetrating ocular injury with extraocular muscle injury by a close-distance blank cartridge that required surgical intervention.

## Case presentation

**Figure 1:**
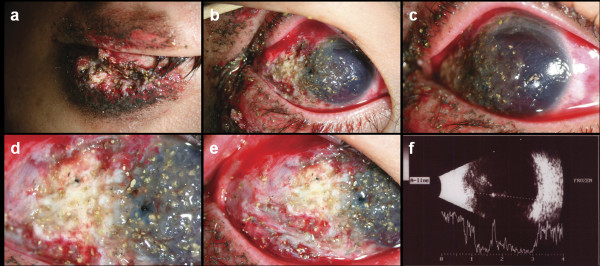
**Anterior segment photography and B-scan ultrasonographic findings of patient. a**, External photography showed a flame burn of the right upper and lower lid with multiple missile wounds of lids. **b**-**e**, Slit lamp examination revealed numerous yellow, powder-like foreign bodies impacted into the ocular surface, severe stromal edema with Descemet's membrane folding, anterior chamber bleeding, lateral rectus muscle splitting injury and traumatic cataract in the right eye. **f**, Transverse view of B-scan ultrasonography showed hyperechoic foreign bodies with mild reduplication echoes and shadowing.

**Figure 2:**
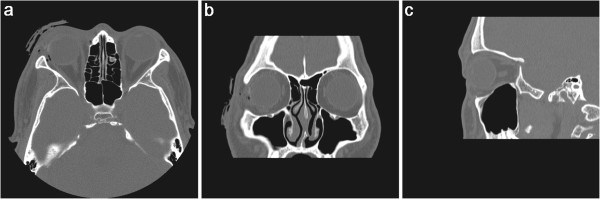
**Unenhanced computed tomography findings of patient. a**-**b**, Axial and coronal view showed accumulation of air near lateral rectus muscle insertion and swelling of preseptal soft tissues in right eye. **a**-**c**, But computed tomography images did not reveal radiopaque foreign body within the right globe.

A 20-year-old man sustained a penetrating globe injury in the right eye while cleaning a blank cartridge pistol. His uncorrected visual acuity at presentation was hand motion and he had a flame burn of his right upper and lower lid with multiple missile wounds of lids, conjunctiva and cornea from close range firing of blank ammunition (Figure 1a).On slit-lamp examination, there was a 12-mm laceration of conjunctiva along the 9 o'clock position with two pinhole-like penetrating injuries of cornea and sclera. There was also a 3-mm corneal laceration between 9 o'clock and 12 o'clock and the exposed lateral rectus muscle was split (Figure 1b-e). Severe Descemet's membrane folding with stromal edema was observed, and numerous yellow, powder-like foreign bodies were impacted in the cornea (Figure 1c). Layered anterior chamber bleeding (hyphema) with traumatic cataract was also noted. The anterior capsule of the lens was ruptured at presentation. The authors could not examine the posterior segment, thus B-scan ultrasonography was performed. Transverse view of ultrasonography showed hyperechoic foreign bodies with mild reduplication echoes and shadowing (Figure 1f). However, a computed tomographic scan using thin section did not reveal a radiopaque foreign body within the right globe (Figure 2).

**Figure 3:**
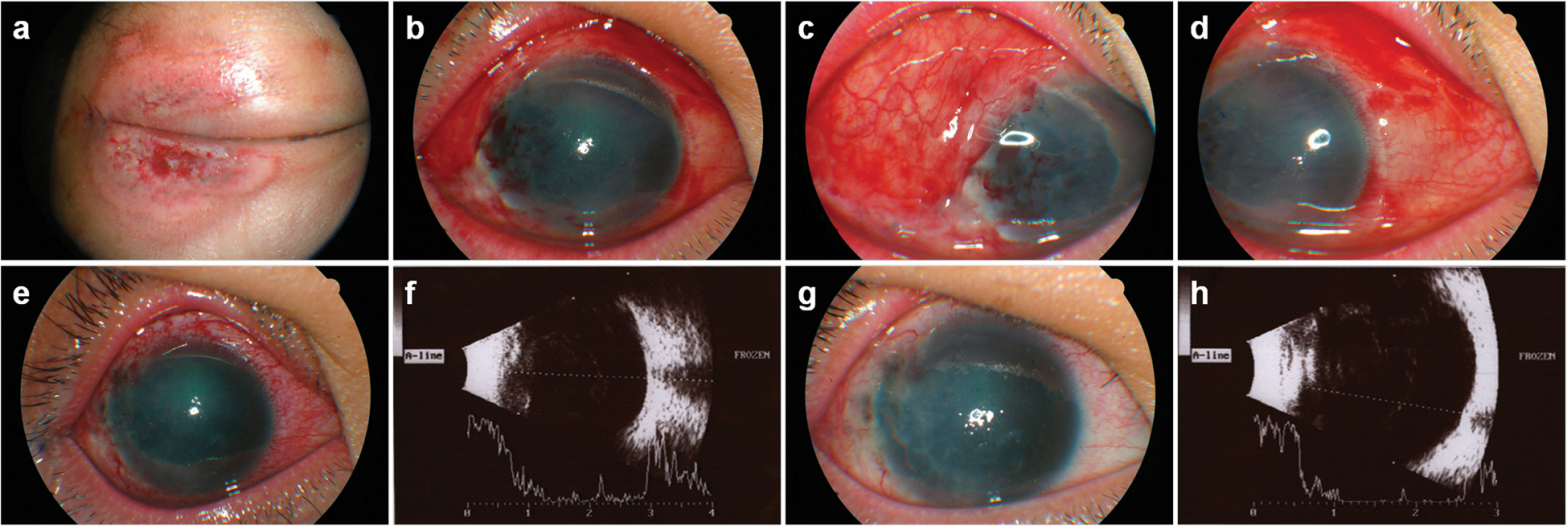
**Postoperative anterior segment photographs and B-scan ultrasonography. a**, External photography showed a 3 cm diameter ring shape abrasion in the upper and lower lids. **b**-**d**, Anterior segment photographs at postoperative day one. **e**-**f**, Anterior segment photograph and ultrasonography at postoperative day 14. **g**-**h**, Anterior segment photograph and ultrasonography at one month after surgery. **f**, **h**, Longitudinal B-scan view showing diffuse low to medium reflective opacities in the vitreous cavity suggesting vitreous hemorrhage.

In the operating room, removal of scattered foreign bodies impacted into corneosclera, tenon, lids, and lateral rectus muscle was performed. Corneoscleral laceration was closed using 10/0 and 8/0 nylon sutures. The split lateral rectus was repaired using 6/0 vicryl sutures, followed by lens aspiration and anterior vitrectomy. Multiple foreign bodies were found in the anterior vitreous during vitrectomy. Necrotic tissue of lids was derided and scrubbed with antibacterial soap, and then canthoplasty was performed. Conjunctival laceration was repaired using 8/0 vicryl sutures. At the end of the surgery, intravitreal vancomycin and ceftazidime were injected to prevent endophthalmitis.At postoperative day 1, intraocular pressure was 7 mm Hg measured by noncontact tonometry, and slit-lamp examination showed the deep anterior chamber without severe inflammation under contact lens (Figure 3a-d). There was no limitation of extraocular movement after surgery, and primary position of the globe was nearly orthophoric. The cornea was gradually stabilized two weeks following surgery. Longitudinal B-scan view showed diffuse low to medium reflective opacities in the vitreous cavity, suggesting vitreous hemorrhage at two weeks after surgery (Figure 3e, f). This patient had corrected visual acuity of counting finger in right eye at one month after surgery. At that time, corneal opacity was denser without inflammation (Figure 3g, h). The authors planned penetrating keratoplasty as further treatment.

## Discussion

This report describes the case of young male patient who sustained penetrating ocular injury with split extraocular muscle by a close-distance blank cartridge shot that required surgical intervention. This case impressively demonstrates the mis-belief that blank cartridges are harmless and relatively safe.

Evaluation of tiny intraocular foreign bodies is problematic in clinical situation. Intraocular foreign bodies undetectable by CT were identified by B-scan ultrasonography in this patient. This finding is opposite to previous study comparing CT, US and MR imaging for ability to demonstrate intraocular glass, CT was shown to be the most sensitive [9]. However, the sensitivity of CT for detecting clinical occult open-globe injuries varied from 56% to 68%, depending on the observer [10]. Glass fragment of 0.5 mm were detected 48% by CT [9], and Zhang et al. [11] reported that CT can fail to detect metal fragments less than 0.5 mm. Moreover, a recent case report describes even 4 mm glass intraocular foreign body that was not identified at 1-mm collimation CT scanning [12]. Considering these reports and this patient, additional B-scan ultrasonography might be helpful to detect small intraocular foreign body when planning the surgical treatment.

Blank cartridge consists of steel cartridge case, freely poured nitrocellulose gunpowder filling and the initial powder charge primer [1,7]. Blank cartridge injuries commonly occur in "mock wars" during Army training [1,2,7]. Previous published articles [7,8] reported that corneal foreign bodies were chemically inert and do not excite an inflammatory reaction. Runyan and Ewald [7] reported that epithelial damage and endothelial edema resolved in 24–72 hours without apparent residual effects. They suggested that blank cartridge injury of the cornea is observed following an explosion generating small missiles with insufficient density or velocity for deep penetration or perforation [7].

**Figure 4:**
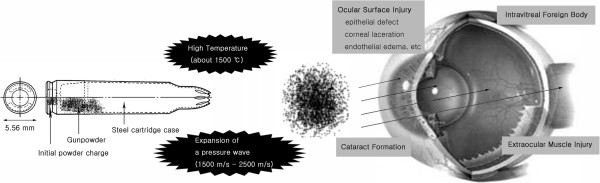
**Mechanisms for ocular damage by blank cartridge.** The authors suggest hypotheses that the blank cartridges firearms demonstrate the severe ocular tissue destruction through two main mechanisms, which consists of baro-trauma and thermal damage.

On the contrary, severe complications including traumatic cataract, split extraocular muscle injury, penetrating globe injury and severe corneal opacity after surgery occurred in this patient. Similarly, Buhner et al. [2] reported a patient with traumatic cataract and iridodialysis caused by blank cartridge injury. To our best knowledge, this is the first case report of extraocular muscle injury with traumatic cataract and penetrating ocular injury caused by blank cartridge injury.The authors suggest hypotheses that the blank cartridges firearms demonstrate the severe ocular tissue destruction through two main mechanisms, which consists of baro-trauma and thermal damage (Figure 4).

Considering baro-trauma, a ignition of 9-mm load will lead to expansion of a pressure wave at 1200 to 1500 m/s, creating 950 mL/g for nitrocellulose and 280 mL/g for black powder. The explosion leads to a pressure 100 to 200 bar at the muzzle of the gun [1]. For a barrel length of 105 mm, a 9-mm load can create a pressure of 5 and 3 bar at a distance of 3 and 5 cm respectively. The powder density in a case may be equivalent to 0.75 and 0.27 J/mm^2^. A projectile has a theoretical capacity to penetrate human skin at minimum value of 0.1 J/mm^2^[13,14]. Even though, close-distance blank cartridge gunshot injury generated powerful energy to make bone defect and penetrating the thoracic wall [1]. The mechanism for occurrence of extraocular muscle splitting injury, penetrating corneoscleral injury and traumatic hyphema was thought be high pressure baro-trauma in this patient. Same mechanisms by baro-trauma (Energy/area density) was proposed in airsoft gun and BB gun injuries [15]. Iris sphincter rupture, corneal rupture, traumatic keratopathy, lens subluxation, and vitreous hemorrhage have been also reported in these injuries [16,17]. Thus, we suggest that the further study for determining the minimum velocity necessary to penetrate the eyes in blank cartridge model is needed.

Besides the direct expanding baro-trauma, counter-coup injury is also important factor in corneal damage. The resultant concussional force is also transmitted to the endothelium though the corneal stroma [7].

The another main mechanism is thermal trauma [15]. The explosion temperature of nitrocellulose is 2500 to 3000'C, which results in a temperature of approximately 1500'C at the muzzle. This high temperatures of burning gas is thought to be cause flame burn in this patient (Figure 1). Moreover, the high temperature might cause formation of CO-hemoglobin, which is evident by bright red muscle tissue [18].

## Conclusions

As a conclusion, blank cartridge guns are dangerous weapons contrary to public opinion. They may inflict potentially fatal injuries to ocular tissue when fired at close-range of fire though baro-trauma and thermal burn. Thus, ophthalmologists should consider the possibility of severe ocular injury by blank ammunition. And additional B-scan ultrasonography should be considered when evaluating eyes with severe trauma associated with blank cartridges.

### Consent

Written informed consent was obtained from the patient for publication of this case report and any accompanying images.

## Competing interests

The authors declare that they have no competing interests.

## Authors’ contributions

SM - participated in information gathering, literature search, data analysis, drafting of the case report, and final approval of manuscript. SHL - conceived the idea, participated in information gathering, literature search, data analysis, drafting of the manuscript, performed the surgery, and approved the final manuscript. Both authors read and approve the final manuscript.

## Pre-publication history

The pre-publication history for this paper can be accessed here:

http://www.biomedcentral.com/1471-2415/14/23/prepub
